# Integrative transcriptome and microbiome analysis reveals ferroptosis-driven duodenal damage caused by Ochratoxin A in mice

**DOI:** 10.3389/fimmu.2026.1804647

**Published:** 2026-04-13

**Authors:** Shaokat Ali, RenZhuo Kuang, Omnia Fathy Abdelkarim, Ali Hassan Nawaz, Muhammad Farhan Rahim, DaoYuan Wang, Ali Asif, MengJin Zhu

**Affiliations:** 1 Key Laboratory of Agricultural Animal Genetics, Breeding, and Reproduction of the Ministry of Education and Key Laboratory of Swine Genetics and Breeding of the Ministry of Agriculture, Huazhong Agricultural University, Wuhan, Hubei, China; 2 The Cooperative Innovation Centre for Sustainable Pig Production, Wuhan, Hubei, China; 3 College of Animal Science and Technology, Nanjing Agricultural University, Nanjing, China; 4 College of Veterinary Medicine, Huazhong Agricultural University, Wuhan, Hubei, China; 5 Reference Laboratory of Veterinary Drug Residues, Huazhong Agricultural University, Wuhan, Hubei, China

**Keywords:** dysbiosis, ferroptosis, microbiome, Ochratoxin A, transcriptomics, transmission electron microscopy (TEM)

## Abstract

Ochratoxin A (OTA), a prevalent mycotoxin produced by fungal contaminants, poses a significant threat to intestinal health. That can induce ferroptosis, a regulated iron-dependent cell death by disrupting duodenal epithelium and gut microbiota homeostasis. We exposed mice to OTA (2 mg/kg body weight/day) for seven days and assessed duodenal damage using histological analysis, transmission electron microscopy (TEM), transcriptomics, quantitative real-time PCR (qRT-PCR), Western blotting, immunofluorescence, biochemical assays, and 16S rRNA sequencing of cecal contents. OTA markedly reduced body weight from day 2 onwards and significantly elevated serum lipopolysaccharides (*LPS*) (P<0.05), duodenal malondialdehyde (*MDA*), and iron levels compared to the control group. OTA significantly diminished duodenal antioxidant defenses, including glutathione, *SOD*, *CAT*, and total antioxidant capacity (*T-AOC*), and caused villus atrophy, crypt hyperplasia, and mitochondrial shrinkage with cristae loss, which are the hallmarks of ferroptosis. Transcriptomic analysis revealed 769 differentially expressed genes (DEGs), including 134 upregulated and 635 downregulated genes, with 26 overlapping ferroptosis-regulating genes (FerroDb). Among these, four key genes *SLC7A11*, *GSTM1*, *CP*, and *SLC40A1* were downregulated, which are major regulators of redox and iron homeostasis, and were enriched in *ROS*/lipid metabolism pathways. Microbiome profiling showed augmented diversification, altered Bacteroidota abundance and enrichment of pathogenic microbiota (e.g., Oscillibacter and Barnesiella), linking ferroptosis with dysbiosis. These findings demonstrate that OTA induces duodenal ferroptosis through dual microbiota-duodenum axis, where microbial dysbiosis amplifies redox imbalance and iron homeostasis. Ferroptotic inhibitors may preserve the gut health in animals and humans exposed to fungal contaminants.

## 1 Introduction

The intestine serves as a crucial interface between the external and internal environment, playing a central role in nutrient absorption, immune regulation, and protection against xenobiotics. Disruption of its integrity, particularly by toxins such as Ochratoxin A (OTA) poses a significant threat to overall health and metabolic homeostasis. OTA is identified as one of the most toxic members of the Ochratoxin family. It has a structural form of a dihyroisocoumarin with a L-phenylalanine, and is a para-chlorophenol compound (1, 2). OTA is among the most dangerous secondary products of fungal of genera *Penicillium* and *Aspergillus* (1, 3, 4). In humans and animals, long-term exposure to OTA in the form of a diet may cause severe toxic effects, such as intestinal, renal, hepatic injury, and immune impairment (5–7). The intestinal barrier is vital for host homeostasis and comprises chemical, physical, immunological, and microbial components. Among these, the physical and microbial barriers are most critical, with the physical barrier primarily maintained by epithelial cells and the overlying mucus layer. (8).

When animals are fed with food contaminated with mycotoxins, intestinal epithelial cells are directly exposed to toxins, resulting in gastrointestinal injury, dysbiosis in microbiome, intestinal dysfunction, malnutrition, poor growth performance, and inflammation (9–11). OTA is a serious and recurring threat to the health of human and animals (12, 13). It has shown an array of toxic responses in diverse animal species in terms of intestinal toxicity, immunotoxicity, teratogenicity and enterotoxicity (14, 15). However, the mechanisms underlying OTA-induced toxicity in humans and livestock remain poorly understood. Comprehensive investigation of its toxicokinetic and toxicological pathways is essential for accurate risk assessment and development of targeted detoxification strategies. OTA exposure typically triggers gastrointestinal dysfunction, inflammation, microbial dysbiosis, and ultimately systemic health deterioration. (7, 11). Numerous diseases were attributed to changes in gut microbiota composition and related pathways (16). Intestinal microbiota has crucial role in toxin degradation process, immune regulation, nutrient absorption, and hormonal metabolism (17–20). Disturbance of the mucosal barrier causes a change in the composition of microbes and translocation, which weakens the host metabolism and immune homeostasis (21, 22). Dysregulation in the structure of microbial community of the host usually referred as dysbiosis (21, 23).

Over the past decades, gut microbiota dysbiosis has been linked to a wide range of pathological disorders and it has been one of the subjects of microbiome studies. The major phyla Firmicutes and Bacteroidetes comprise most intestinal microbial community and their relative proportion is one of the primary signs of intestinal health (24, 25). Alterations in microbiota composition, abundance, and function exert both local and systemic effects, potentially modifying physiology and disease susceptibility through diffusible metabolites that interact with distant organs. (26–29). The principal gut microbial metabolites include short-chain fatty acids (*SCFAs*) (30, 31), bile acids (*BAs*) (31), lipopolysaccharides (*LPS*) (32), trimethylamine N-oxide (*TMAO*) (33), succinate (34), and amino acids (35). Gut microbial ecosystem dysregulation undermines the integrity of the gastrointestinal barriers and systems inflammation, which allows bacteria and metabolite translocation to the intestinal tissues (36, 37).

New data suggest that intestinal microbiota has profound regulatory roles on the host inflammation systems and immune system, especially the intestinal immunity (38). The current trends in microbiology, immunology, and oncology have generated significant interests in regulatory action of gut transcriptome, gut microbiome, their combination, and in metabolites in ferroptosis regulation (39). Ferroptosis is a recent cell death pathway that has been specifically identified to be a fundamental role in the pathophysiology of many different diseases in humans and animals (40). It has been noted, ferroptosis interferes with composition and metabolic activity of gut microbiota, which add to intestinal injury (41). On the other hand, ferroptosis can be controlled by microbial metabolites, community structures, and iron homeostasis that are regulated by the gut microbiota in case of dysbiosis (42). Iron is an essential trace element in the health of humans and animals and could also be associated with such disorders as hemophilia, aging, sickle cell disease, and thalassemia (43, 44). Increased intracellular iron and lipid peroxidation also have significant contribution to oxidative stress tissue damage (45, 46).

Ferroptosis is a regulated cell death that is based on iron accumulation and is non-apoptotic; it is overloaded with lipid peroxidation (47, 48). It mostly occurs due to the impairment of the iron homeostasis and reactive oxygen species (*ROS*) generation (F. 49–51). Ferroptosis is caused by the iron-catalyzed *ROS* production through the Fenton reaction (52). It has been demonstrated that this action leads to inflammation, dysfunction of the intestinal system, and dysregulation of gut microbes (53, 54). Ferroptosis is also linked with the maladaptive accumulation of *ROS* (55), the catabolism of glutathione (*GSH*) malfunction, transport dysfunction of cysteine, and endoplasmic reticulum stress (56, 57). Morphological features consist of the shrinkage of mitochondria, loss of cristae, high density of membrane, and rupture of outer membrane(X. 49, 51, 58). Solute carrier family 7 member 11(*SLC7A11*) is a regulatory ferroptosis-critical protein that is a cell membrane localized protein. *SLC7A11* malfunctioning contributes to accretion of lipid peroxides and consequent breaking apart of plasma membrane, and as a consequence, *SLC7A11* qualifies as a significant determinant of ferroptotic vulnerability (16). It has been shown that *GSH* and cysteine are essential to the survival of a cell under the state of redox stability. *GSH* is a very strong antioxidant that neutralizes the effects of lipid peroxidation, and its loss increases the vulnerability of cells to ferroptosis (59). Alteration of the intestinal immune system triggers inflammation and impairs the physiological integrity. The commensal microorganisms constitute the biological barrier and it is the major organism that regulates intestinal physiological processes (60). We hypothesized that OTA induces duodenal ferroptosis driven damage in mice by disrupting iron homeostasis (Increased labile iron pool) as down-regulation of SLC40A1/FPN and CP that is validated through western blotting and m-RNA expression. While oxidative stress was also triggered (elevated MDA/Fe, reduced GSH/SOD) that was evidenced by TEM mitochondrial changes and histology. This study aims to investigate the impact of OTA on intestinal integrity and homeostasis by performing a combined analysis on ferroptosis-mediated cell death using dual duodenal transcriptomic and microbiome of the gut. In particular, this study tends to elucidate how the gut microbiota-duodenum axis contributes to ferroptosis-mediated intestinal injury in mice exposed to OTA. The findings indicate that OTA promotes ferroptotic intestinal damage by disrupting iron homeostasis and redox balance in close association with gut microbiota dysbiosis.

## 2 Material and methods

### 2.1 Material and reagents

This study utilized antibodies and chemical reagents from multiple commercial suppliers. OTA (CAS 303-47-9) was sourced from Sigma-Aldrich (USA), and dimethyl sulfoxide (*DMSO*, catalogue BL1114B) was acquired from Biosharp (Beijing, China). Reverse transcription kits (RR047A) were obtained from Takara (Japan), and *SYBR* Green reagents and BCA protein quantification kits (E112-02) were sourced from Vazyme (China). RIPA buffer was obtained from Servicebio (Wuhan, China).The study employed a rabbit *Anti-SLC7A11* (A13685, ABclonal, China), Rabbit *Anti-GSTM1* (A17492, ABclonal, China), Rabbit anti-Ceruloplasmin *CP*(A20229,ABclonal,China), Rabbit *Anti-SLC40A1/FPN*(A14885,ABclonal,China) and Alexa Fluor 488-tagged goat anti-rabbit IgG (GB25303, Servicebio, China), and goat anti-rabbit IgG H&L (Abcam, ab6702). The secondary antibody was goat anti-rabbit HRP IgG (AS014; ABclonal, China). Total Iron ion Assay kit (G4301), Total antioxidant capacity assay kit *T-AOC* (G4312), Malondialdehyde (*MDA*) assay kit (G4302), Catalase (*CAT*) activity assay kit (G4307), Total superoxide dismutase (*T-SOD*) activity assay kit (G4306), and Reduced Glutathione (*GSH*) assay Kit (G4305) were obtained from Wuhan, Servicebio Technology Co., Ltd.

### 2.2 Animal model and experimental design

Male C57BL/6 mice aged 7–8 weeks were kept in a controlled environment at the Experimental Animal Center of Huazhong Agricultural University. The room was maintained a 12-hour light/dark cycle, with a temperature of 22 ± 2 °C and humidity of 55 ± 5%. The mice had free access to water and standard diet. This study was approved by the Animal Experiment Ethics Review Committee of the Experimental Animal Center of Huazhong Agricultural University (Ethics ID: HZAUMO-2025-0363). The mice were randomly assigned to two groups, each consisting of ten mice. Prior to treatment, all mice underwent a 7-day acclimatization period in an experimental environment to minimize stress and variability. Starting on day 8, the control group was administered intragastric doses of *DMSO* as a vehicle control, whereas the treatment group received OTA at a dose of 2 mg/kg body weight daily for seven consecutive days. On day 14, all mice were euthanized, and their serum and intestinal tissues were promptly collected and preserved in liquid nitrogen for subsequent analysis.

### 2.3 Biochemical assays and LPS enzyme linked immunosorbent assay

Following OTA treatment and control with *DMSO* treatment, the levels of *MDA* in duodenal tissues, total antioxidant capacity(*T-AOC*), glutathione activity (*GSH* level), catalase activity (*CAT*), total superoxide dismutase level(*T-SOD*), and duodenal iron level (Fe level) were assessed according to the manufacturer’s guidelines (Servicebio, Wuhan, China). Briefly, Duodenal tissues (~50–100 mg) from each sample were homogenized in ice-cold PBS (1:10 w/v) using metallic beads in a tissue disruptor (3 × 30 s cycles at 60 Hz). Homogenates were centrifuged at 12,000 × g for 10 min at 4 °C, and supernatants were collected for assays. All biochemical kits were from Servicebio (Wuhan, China) and performed per manufacturers’ instructions (Servicebio G4301–G4307 manuals). To detect total iron assay (G4301) tissue homogenate (20μL) mixed with chromogenic agent, incubated 30 min at 37 °C, absorbance at 550 nm. Fe content calculated vs. standard curve (μmol/g tissue). For Malondialdehyde (MDA, G4302) tissue homogenate reacted with Thio barbituric acid (TBA) at 100 °C for 60 min, cooled, centrifuged; supernatant absorbance at 532nm. MDA reported as nmol/mg protein. While for Reduced glutathione (GSH, G4305) tissue homogenate mixed DTNB reagent from kit incubated at 25 °C for 5 min, absorbance 412nm. GSH as U/mg protein. Furthermore, for total superoxide dismutase (T-SOD, G4306, WST-1 method) tissue homogenate and enzyme working solution mixed with WST-1 incubated at 37 °C for 20–30 min absorbance was measured at 450 nm. Inhibition rate calculated, activity measured as U/mg protein. Moreover, for Catalase (CAT, G4307) tissue homogenate dissolve in H_2_O_2_ substrate, incubated at 25 °C for 10 min absorbance was measured at 520 nm. CAT measured as U/mg protein. For total antioxidant capacity (T-AOC, G4312, FRAP method) tissue homogenate and Fe³^+^-TPTZ incubated 37 °C 5min and absorbance was measured at 593 nm vs. Fe²^+^ standards (U/mL). Protein concentrations determined by BCA (Vazyme E112-02) for normalization. For ELISA, blood samples were collected and left at room temperature for 1h to allow coagulation and then centrifuged at 3000 × g for 15 min at 4°C. The supernatant was carefully collected and stored at -20 °C. LPS levels were determined using a kit (SEB526Ge, Cloud-Clone Corp). Briefly,100μL serum/standards added to pre-coated plate (37 °C, 1 h), Detection Reagent A (100× dilution, incubate at 37 °C for 1 h, wash 3×), Detection Reagent B (100× dilution, 37 °C, 30 min, wash 5×), TMB substrate (Incubate at 37 °C for 10–20 min), stop solution. Concentrations interpolated from 4-parameter standard curve (0.47–30 ng/mL range). The results for *LPS* were measured at OD 450nm using microplate reader.

### 2.4 Histopathology(H&E) and immunohistochemistry

To evaluate the histological parameters post-treatment, fresh duodenal tissues were rinsed with PBS to eliminate blood and then preserved in 4% paraformaldehyde (G1101, Servicebio). Paraffin-embedded sections were dewaxed by sequential immersion in environmentally friendly transparent de-waxing liquids I and II for 20 min each. This was followed by dehydration using anhydrous ethanol I and II for 5 min each, followed by 75% ethanol for 5 min, and finally a rinse with tap water. The sections were pretreated with HD constant staining solution for 1 min, stained with hematoxylin for 3–5 min, differentiated, blued, and rinsed with tap water. Eosin staining was conducted using 95% ethanol for 1 min, followed by 15 s in eosin dye. The dehydration process involved immersing the sections in absolute ethanol I–III, normal butanol I–II, and xylene I–II, each for 2 min, before mounting with neutral gum. The stained sections were examined under a microscope for image capture and analysis. The cell nuclei were blue, and the cytoplasm was stained red. For immunohistochemistry, the tissues were fixed in 4% paraformaldehyde and underwent three rounds of deparaffinization using a xylene substitute for 10 min each. They were rehydrated with a series of ethanol solutions, rinsed with distilled water, and sectioned as required. To inhibit endogenous peroxidase activity, a 3% hydrogen peroxide solution was applied for 25 min at room temperature, followed by a PBS rinse. To prevent non-specific binding, 3% BSA was used for 30 min, tailored to the primary antibody source. The samples were then treated with *anti-4-HNE* (GB150073, Servicebio, Wuhan, China) and subsequently with HRP-conjugated anti-rabbit IgG for 50 min at room temperature. DAB was freshly prepared for color development, observed under a microscope until brown staining was visible, and rinsed with tap water to halt the reaction. Hematoxylin was used to counterstain the nuclei for 3 min, followed by brief differentiation and bluing under running water. Finally, the sections were dehydrated using graded ethanol and xylene, mounted with neutral resin, and examined under a microscope. Images were captured using a digital microscope camera (Olympus BX53) and the Cell Sense software.

### 2.5 Transmission electron microscopy of duodenum tissue

Fresh duodenum tissue samples were collected within 1–3 min, avoiding mechanical damage, immediately immersed in TEM fixative, trimmed into 1 mm3 pieces within the fixative, and stored at 4 °C overnight. Post-fixation, samples were treated with 1% osmium tetroxide (OsO_4_) in 0.1M Phosphate buffer PB (PH 7.4) for 2 h at room temperature in the dark, followed by three 15 min PB washes. Dehydration was carried out sequentially in 30%,50%,70%,80%,95% and 100% ethanol for 20 min each and twice in 100% acetone for 15 min. Samples were infiltrated with an acetone:812 embedding medium mixture (1:1 for 2–4 h at 37°C, then 1:2 overnight), followed by pure 812 resin infiltration at 37°C for 5-8h. The specimens were embedded in fresh resin and polymerized at 60°C for 48h. Semi-thin sections (1.5µm) were stained with toluidine blue for orientation, and ultrathin sections (60–80 nm) were cut using an ultramicrotome and mounted on 150-mesh copper grids. The sections were stained with 2% uranyl acetate in saturated ethanol for 8 min in the dark, washed with 70% ethanol and ultrapure water, and stained with 2.6% lead citrate for 8 min. After washing and air-drying overnight, the samples were examined and imaged using a transmission electron microscope.

### 2.6 Transcriptomic sequencing and data analysis

Total RNA was isolated from duodenal tissues using TRIzol reagent (Qiagen), followed by mRNA purification with magnetic beads attached to poly T oligos. The extracted mRNA was then broken into smaller pieces and converted to cDNA through reverse transcription. The cDNA was subsequently amplified and cleaned using the AMPure XP system (Beckman Coulter, USA) to produce 250–300 bp fragments. The purity and integrity of the total RNA were assessed using an Agilent 2100 Bioanalyzer, considering RIN values above 7.00 as acceptable for total RNA. Library preparation was performed using the Hieff NGS Ultima Dual-mode mRNA Library Prep Kit for Illumina (Cat. No. 13533ES96). For purification, Hieff NGS DNA Selection Beads (Yeasen, Cat. No. 12601ES56) were used as efficient alternatives to the Ampure XP beads. The libraries were sequenced as 150 base pair paired-end reads on the Illumina NovaSeq 6000 platform. Raw reads were processed using custom Perl scripts to eliminate adapter sequences, poly-N regions, and low-quality bases. RNA-seq FASTQ reads were mapped to the mouse reference genome (mm10) using the STAR software (2.7.5c). The read counts for each gene were calculated with the RSEM software suite (version 1.3.3). A differential analysis was conducted between the treatment and control groups. The read count matrix served as the input for the edgeR and Limma algorithms in R v4.5.0. A gene was considered expressed if it had a count per million (CPM) exceeding 1 in at least one of the treatment groups. The CPM (expression matrix) was used to perform correlation and Principal Component Analysis (PCA) in R v4.5.0. To investigate the functional annotation of differentially expressed (DEGs) for GO and KEGG enrichment pathways, the DAVID database webtools were utilized. Significant GO and KEGG pathways associated with ferroptosis were visualized using R v4.5.0 for bubble charts.

### 2.7 RNA extraction and qRT-PCR

RNA from intestinal tissues was isolated using the Cell Fast RNA Extraction kit for Animals (RK30120, ABclonal) following the manufacturer’s instructions. The purity and concentration of the RNA were measured using a NanoDrop-2000 (Thermo Fisher Scientific, USA). RNA was reverse transcribed using the PrimeScriptTM RT reagent kit with gDNA Eraser (Perfect Real Time) (Takara, cat#RR047A, Japan). Quantitative real-time PCR was conducted with Taq Pro Universal SYBR qPCR Master Mix (Q712, Vazyme). Gene expression was evaluated using the 2-ΔΔCT method, with GAPDH as the internal reference gene.

### 2.8 Western blotting

Total protein was extracted from tissues that were homogenized using metallic beads and combined with RIPA buffer, a phosphatase inhibitor, a protease inhibitor, and a 50 × PMSF cocktail. The mixture was subsequently centrifuged at 4 °C at 12000×g for 10 min, and the supernatant was collected as the total protein. The BCA protein assay (Vazyme) was used to quantify total protein. Proteins were denatured in an SDS-loading buffer at a 5:1 ratio of protein to loading buffer and incubated at 95 °C for 5 min before being loaded onto the gel. Between 20-40μg of protein from each group was applied to a 10% Tris-glycine sodium dodecyl sulfate polyacrylamide gel electrophoresis gel, maintained at a constant voltage of 80–100 V. The gel was then transferred to a polyvinylidene difluoride (PVDF) membrane using a constant amperage current of 200 mA with a protein transfer apparatus (Trans-Blot Turbo; Bio-Rad, USA). Once the protein was transferred to the PVDF membrane, it was blocked with 5% skimmed milk in TBST for two hours at room temperature. The membrane was washed three times with TBST and incubated with anti-rabbit primary antibodies for protein used in this study at 4 °C overnight, following the manufacturer’s instructions for antibody dilutions. After primary incubation, the PVDF membrane was washed three times with TBST. Subsequently, the membrane was incubated with horseradish peroxidase-conjugated anti-rabbit secondary antibody at room temperature for two hours. Following secondary antibody incubation, the membranes were washed three times with TBST. The protein bands on the PVDF membrane were visualized using an enhanced chemiluminescence solution (Biosharp, Guangzhou, China). Protein expression was analyzed using ImageJ v1.54p, with normalization to β-actin as the internal control.

### 2.9 Immunofluorescent analysis

Duodenal tissue sections embedded in paraffin were first deparaffinized and then rehydrated using a series of ethanol solutions. Antigen retrieval was then performed. After rinsing with PBS (pH 7.4), the sections were blocked with 3% BSA for 30 min and then incubated with the primary antibody at 4 °C overnight. Following PBS washes, secondary antibodies were applied for 50 min at room temperature in the dark. Nuclei were counterstained with DAPI for 10 min, and autofluorescence was minimized using quencher B for 5 min. Slides were mounted with an anti-fade medium, and images were captured at the following excitation/emission wavelengths (nm): DAPI 330–380/420, FITC 465–495/515–555, CY3 510–560/590, and CY5 608–648/672–712.

### 2.10 Gut microbiota sequencing and data analysis

The gut microbiome was analyzed using cecal content for 16S RNA sequencing. Upon completion of the animal experiments, cecal contents were promptly collected in sterilized EP tubes, immediately stored in liquid nitrogen, and later stored at -80 °C. Subsequently, samples were forwarded for DNA extraction and microbiome sequencing by BMK utilizing Illumina Novaseq Platform. The amplicon 16s V3+V4 regions were sequenced after DNA extraction and sequencing was conducted on an Illumina Novaseq 6000 platform with paired ends (PE) assembled using USEARCH (version 10) to ensure quality control. The primer sequences were used in this study as follows: forward: ACTCCTACGGGAGGCAGCA; reverse: GGACTACHVGGGTWTCTAAT. High-quality reads generated from the above steps were used in downstream analyses. Sequences with similarity ≥ 97% were clustered into the same operational taxonomic unit (OTU) using USEARCH (v10.0), and OTUs with abundance <0.005% were filtered. Taxonomic annotation of the OTUs was performed based on the Naive Bayes classifier in QIIME2 using the SILVA database (release 138) with a confidence threshold of 70%. Alpha diversity was calculated and displayed using QIIME2 and R software, respectively. Beta diversity was determined to evaluate the degree of similarity between microbial communities from different samples using QIIME. Principal coordinate analysis (PCoA), heat maps, UPGMA and nonmetric multidimensional scaling (NMDS) were used to analyze the beta diversity. Furthermore, we employed Linear Discriminant Analysis (LDA) effect size (LEfSe) to test the significant taxonomic differences among groups. A logarithmic LDA score of 4.0 was set as the threshold for the discriminative features. To explore the dissimilarities in the microbiome among different factors, a redundancy analysis (RDA) was performed in R using the ‘vegan’ package.

### 2.11 Statistical analysis

Statistical data analysis was conducted using two separate software tools: GraphPad Prism 9.0 and R software. All quantitative data are presented as mean ± standard error of the mean (SEM) with three independent replicates. The statistical analyses included a range of tests, either t-test or one-way ANOVA. Differences were considered statistically significant at p<0.05. For transcriptomic data differential analyses were performed using the edgeR and Limma packages in R. The Benjamini-Hochberg procedure was applied for multiple testing corrections, with a false discovery rate (FDR) threshold set at 0.05. These analyses were performed across various groups, with significant differences between groups identified based on p-values <0.05.

## 3 Results

### 3.1 OTA reduces growth performance, induce oxidative stress and iron accumulation in duodenum

**Figure 1:**
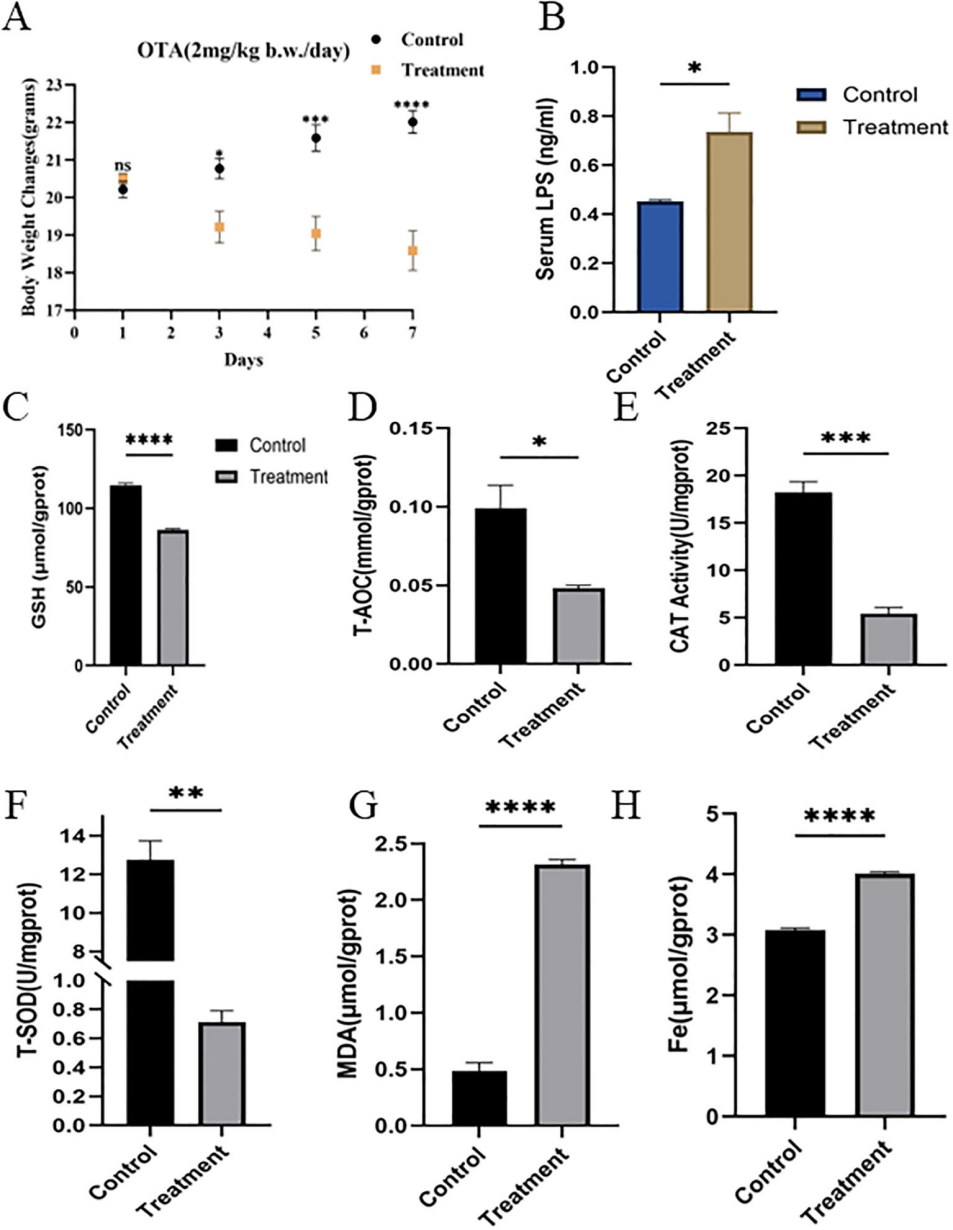
OTA reduces growth performance and disrupts redox and iron homeostasis in mice **(A)** Effect of OTA on body weight after 7-day exposure **(B)***LPS* level in duodenal tissues as measured by ELISA **(C-F)** Duodenal redox status upon exposure to OTA **(G)***MDA* level in duodenal tissues **(H)** Iron level in duodenal tissues subjected to OTA treatment. All data are presented as mean ± SEM(n=3), with statistical significance indicated as follows: ^ns^P>0.05, *P<0.05, **P<0.01, ***P<0.001, ****P<0.0001.

C57BL/6 mice were treated with OTA for 7 days to investigate its impact on duodenal tissues. The duodenum represents the first line of defense against dietary contaminants and is susceptible to ferroptosis induction. **Figure 1** demonstrates distinct biochemical and physiological changes in the OTA treatment group compared to controls. Body weight decreased significantly (P<0.05) from day 2 through day 7 in OTA-treated mice, while control mice gained weight, indicating systemic toxicity (**Figure 1A**). In contrast, biochemical assays conducted on duodenal tissues and lipopolysaccharide levels in serum provide mechanistic insights. The *LPS* levels in blood serum after treatment were measured, and the results confirmed that the *LPS* level was significantly (P<0.05) higher in the OTA-treated groups. Elevated *LPS* levels indicate inflammation in the gut, which can subsequently increase systemic oxidative stress (**Figure 1B**). Simultaneously, glutathione activity (*GSH*) (**Figure 1C**), total antioxidant capacity (*T-AOC)* (**Figure 1D**), catalase activity (*CAT*) (**Figure 1E**) and total superoxide dismutase (*T-SOD*) (**Figure 1F**) were all significantly(P<0.05) reduced, reflecting the impairment of antioxidant defenses that protect against lipid peroxidation and ferroptosis. These changes strongly suggest that OTA disrupts redox homeostasis in duodenal tissues, promoting an environment conducive to ferroptosis in the OTA treatment group compared to the control group. Moreover, OTA significantly increased (P<0.05) malondialdehyde (*MDA*) (**Figure 1B**) and iron levels (**Figure 1H**) in duodenal tissues, indicating oxidative stress damage and ferroptosis in tissues following OTA treatment. In particular, increased *MDA* and Iron levels, increased Serum *LPS* levels, and suppressed *GSH, SOD, T-AOC,SOD*, and *CAT* activities collectively contribute to ferroptotic cell death and loss of tissue function.

### 3.2 OTA induces histological damages and lipid peroxidation in mice duodenum

**Figure 2:**
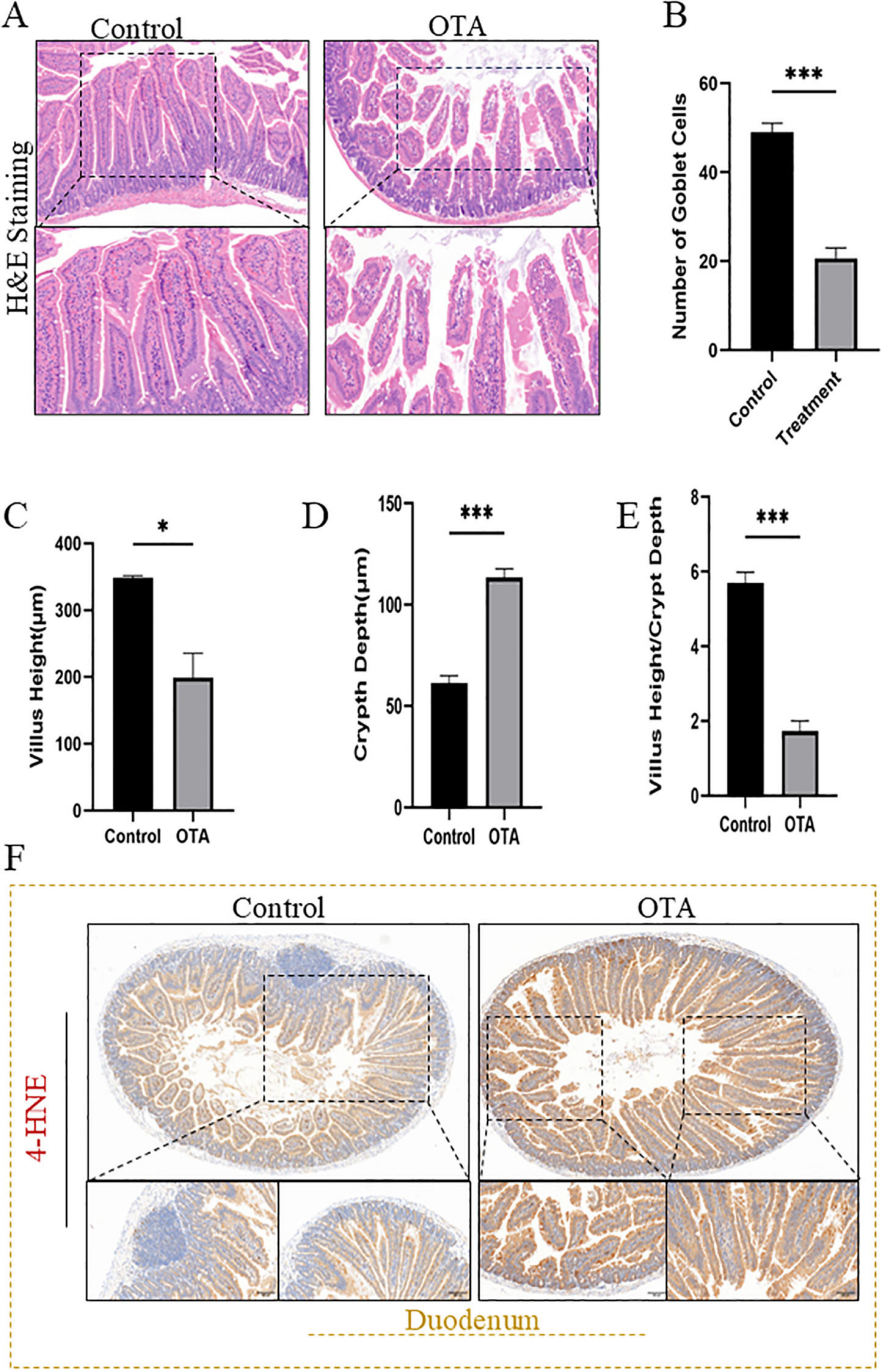
OTA induces histopathological changes in the duodenal tissue of mice. **(A)** H&E staining of the duodenal tissue sections. **(B)** Number of Goblet cells per duodenal villus. **(C-E)** Quantification analysis of duodenal villus height, crypt depth, and ratio of villus height/crypt depth. **(F)**Immunohistochemistry for 4-HNE, a ferroptosis marker, in duodenal tissue, indicating higher levels of *4-HNE* in the treatment group than in the control group exposed to OTA. All data are presented as mean ± SEM(n=3), with statistical significance indicated as follows: *P<0.05, ***P<0.001.

Comparing the duodenal histological sections of the treatment and control groups, it was evident that OTA induced a significant change in villus density, villus height, and depth of the villus crypt. In contrast, the number of goblet cells per villus was also affected by the number and morphological changes in epithelial enterocytes, and the integrity of the mucosal and muscular layer connections were also disturbed, as shown in **Figure 2A**. OTA treated group had fewer goblet cells per villus than the control group (**Figure 2B**) indicating disruption of the defense and immune function of duodenal tissues induced by OTA. In addition, OTA treatment caused a reduction in villus height compared to the control group (**Figure 2C**), while a significant increase in crypt depth (**Figure 2D**) was observed, thereby reducing the villus height/crypt ratio in the treatment group (**Figure 2E**). These changes disrupt the structural and functional integrity of the duodenum. Furthermore, we observed that this histological damage was due to apoptotic cell death or ferroptosis. Therefore, we performed immunohistochemistry for the prominent ferroptosis marker *4-HNE*(4-Hydroxyneonal) as shown in **Figure 2E**. *4-HNE* is a well-established marker of lipid peroxidation associated with ferroptotic cell death. The OTA-treated group displayed extensive *4-HNE* positivity compared to the control group, indicating elevated lipid peroxide levels, oxidative damage, and membrane lipid degradation in the duodenal tissue.

### 3.3 Transmission electron microscopy and transcriptomic profiling identify oxidative stress and ferroptosis associated signatures

**Figure 3:**
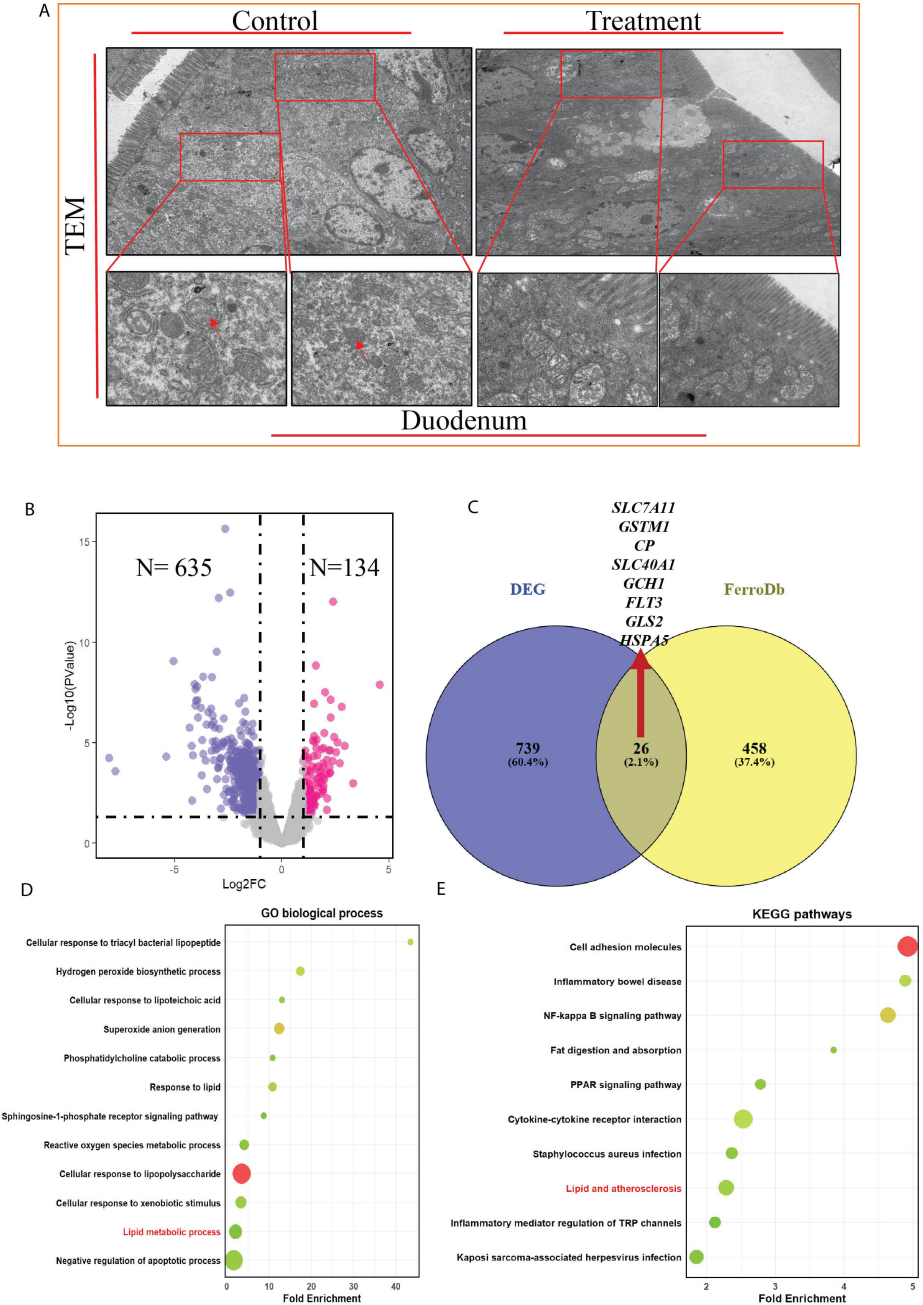
OTA effect on transmission electron microscopic histology and on transcriptome of duodenal tissues**(A)** Representative TEM images of duodenal enterocytes showing morphological characteristics of ferroptosis in OTA treated samples such as condensed mitochondrial membranes, cristae diminished, damaged endoplasmic reticulum as well as damaged enterocyte membrane indicated with red arrows in control and treatment group **(B)** Volcano plot indicating DEGs in treatment group compared to control with 635 up-regulated and 134 down-regulated Log2FC>1, P<0.05, (n=3) **(C)** Venn Diagram for DEGs from transcriptome of duodenal tissue and Ferroptosis database(FerroDb) gene indicating 26 overlapping genes that regulate ferroptosis in duodenal tissue **(D)** Gene ontology enrichment analysis for biological process for DEGs (n=3) **(E)** DEGs KEGG pathways enrichment analysis for duodenal tissues,(n=3).

To investigate effect of OTA on duodenal enterocytes, we used transmission electron microscopy to reveal cellular organelle damage. TEM observation (**Figure 3A**) of enterocyte revealed there is cell membrane damage in treatment group compared to control group. Furthermore, TEM observation revealed condensed mitochondria, damaged endoplasmic reticulum, and vanished or damaged cristae of mitochondria of enterocytes, as indicated by the red arrow in **Figure 3A**. These are typical lesions associated with ferroptotic cell death. To evaluate the transcriptomic changes in duodenal tissue to reveal OTA toxicity, mRNA sequencing analysis was performed on duodenal tissues from the control and treatment groups. The quality and mapping data are mentioned in **Supplementary Table 1**. OTA treatment genes were identified in the duodenal transcriptome DEGs. Compared with the control, there were 769 DEGs, of which 635 were significantly downregulated with a fold change greater than 1 (P<0.05), and 134 were upregulated, as shown in the volcano plot (**Figure 3**). B. To identify genes associated with ferroptosis, DEGs from transcriptomic data were combined with Ferroptosis database (FerroDb) genes, from which we identified 26 genes associated with ferroptosis, as shown in the Venn diagram (**Figure 3C**). Among these eight redox-regulating genes, iron homeostasis was a key marker for inducing ferroptosis in the duodenum in the treatment group. The genes *SLC7A11, GSTM1, CP, SLC40A1/FPN, FAR1, GLS2, FLT3*, and *HSPA5* were identified. Among them, only *HSPA5* was upregulated as a compensatory response to ferroptosis, while others were downregulated, indicating dysregulation of iron and redox homeostasis, as previously observed in redox and iron levels in tissues. Furthermore, GO and KEGG enrichment analyses were conducted for DEGs. Biological process GO is illustrated in **Figure 3D** (**Supplementary Table 3**) in which response to hydrogen peroxides, superoxide, lipopolysaccharides, response to lipids, and lipid metabolic processes were enriched, indicating ferroptosis in duodenal tissue. Meanwhile KEGG (**Supplementary Table 3**) enrichment analysis showed that main pathways supporting ferroptosis induction are fat digestion and absorption, *PPAR* signaling, lipid arteriosclerosis, and *NF-Kβ* signaling pathways (**Figure 3E**).

### 3.4 OTA induced ferroptosis associated marker functional validation at m-RNA and protein level

**Figure 4:**
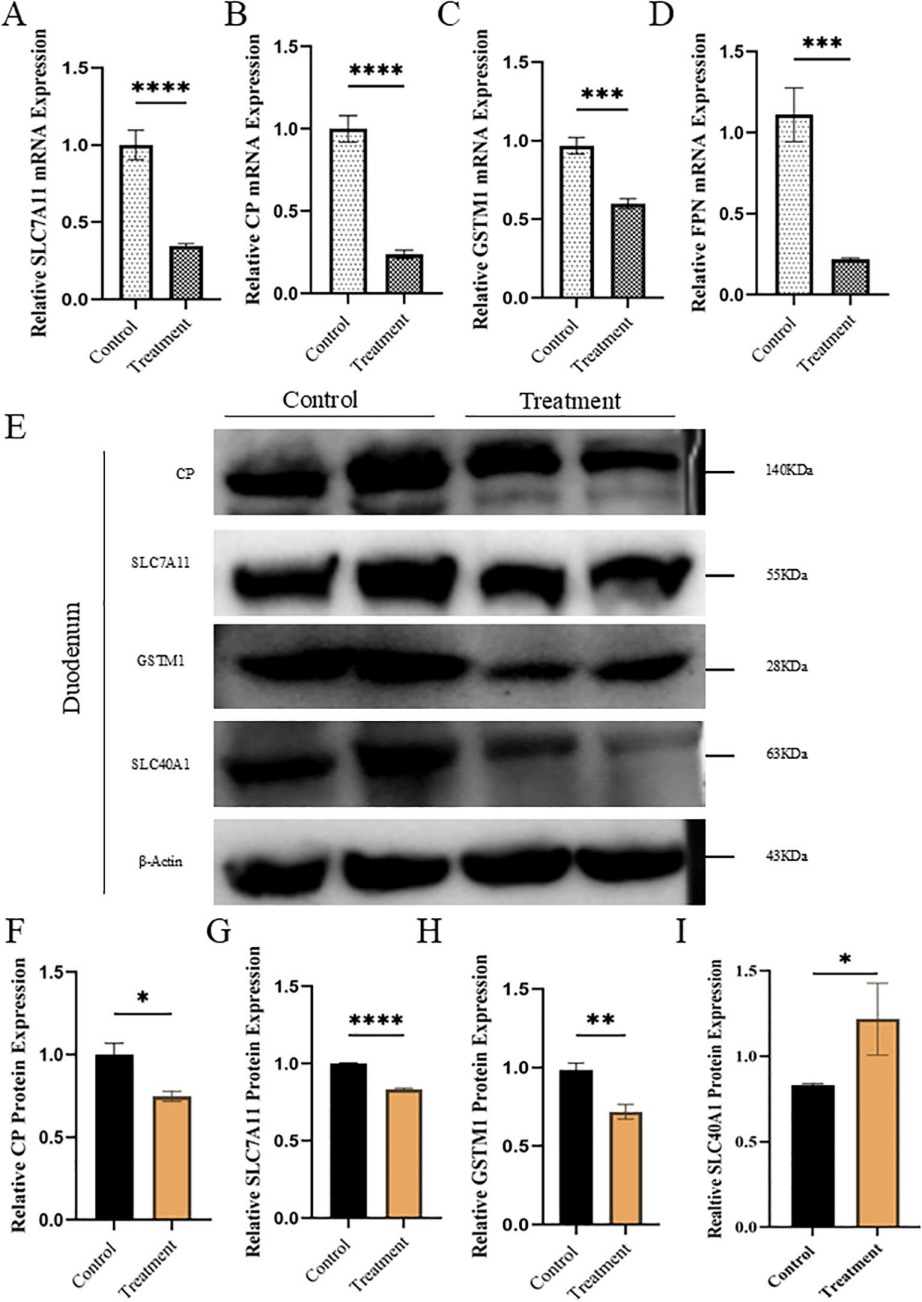
Functional validation of genes associated with ferroptosis in duodenal tissue exposed to OTA **(A-D)** Relative mRNA expression of *SLC7A11,CP,GSTM1*, and *SLC40A1/FPN*, the main ferroptosis markers involved in duodenal injury **(E)** Representative images of western blotting bands for proteins associated with ferroptosis-like injury in the duodenum **(F-H)** Relative protein quantification for *SLC7A11,CP,GSTM1* and *SLC40A1/FPN* in duodenal tissues involved in ferroptosis pathways. All data are presented as mean ± SEM(n=3), with statistical significance indicated as follows: *P<0.05, **P<0.01, ***P<0.001, ****P<0.0001.

**Figure 5:**
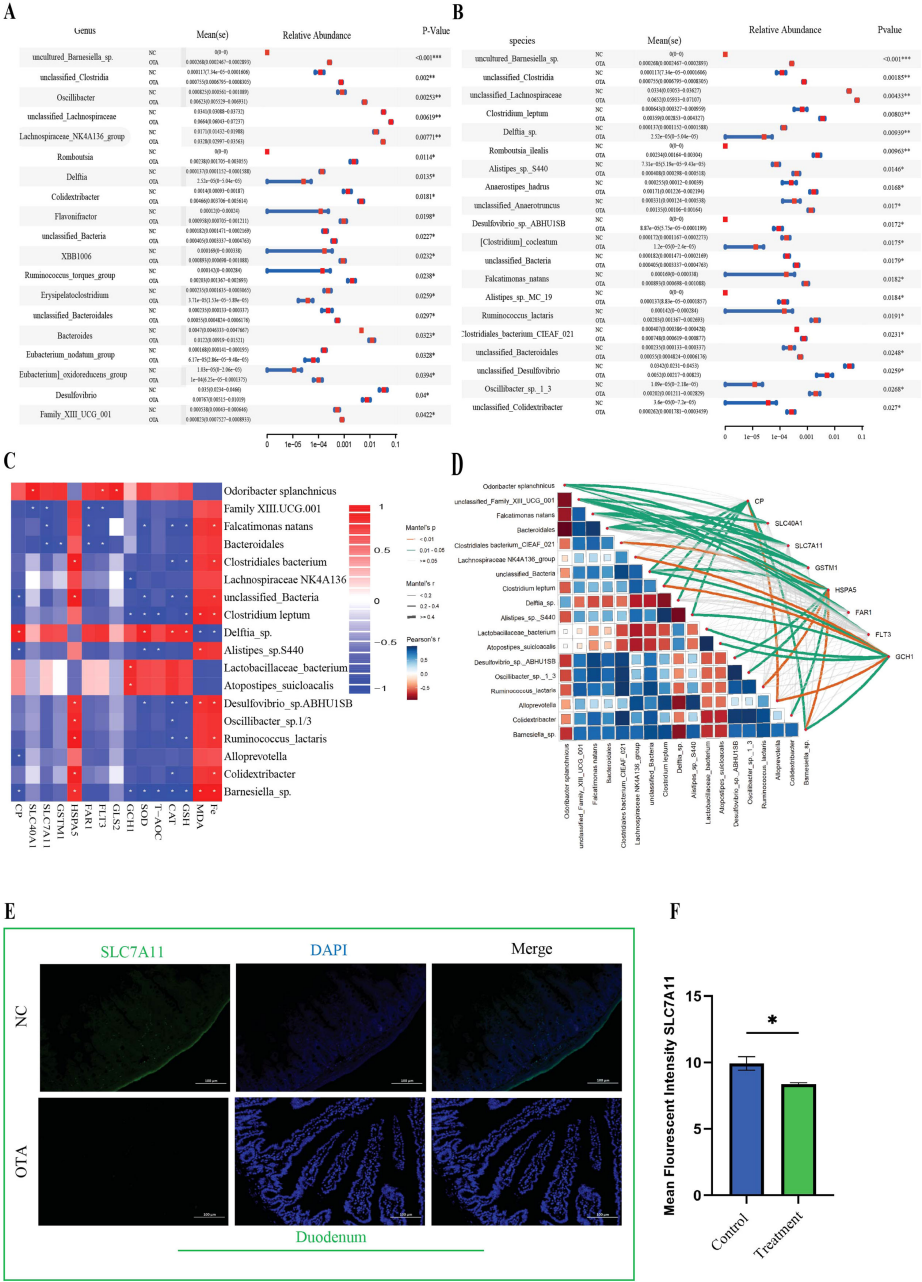
Integrated analysis of gut microbiota and duodenal transcriptomic data **(A-B)** STAMP analysis for differential abundance of gut microbial genera and species. **(C)** Pearson correlation between redox parameters and ferroptosis-regulating differential genes with significantly differential microbial species abundance in the gut. **(D)** Mantel’s correlation between redox parameters and ferroptosis-regulating differential genes with significantly differential microbial species abundance in the gut microbiome. **(E, F)** Representative image of immunofluorescence for *SLC7A11* protein in duodenal tissues and quantification analysis. All data are presented as mean ± SEM(n=3), with statistical significance indicated as follows: ^ns^P>0.05, *P<0.05, **P<0.01, ***P<0.001, ****P<0.0001.

Transcriptomic profiling, along with assessment of iron concentration and redox-antioxidant status in duodenal tissues, revealed clear evidence of ferroptotic cell death. This initial observation was further corroborated by both mRNA and protein expression analyses. To validate these findings, we selected four key genes, *SLC7A11, CP, GSTM1*, and *SLC40A1(FPN*), from the eight core ferroptosis-related markers for further examination at the transcriptional level. The primers used for these genes are given in **Supplementary Table 2**. As shown in Figures A-D, the mRNA expression levels of all four genes were significantly reduced in the treatment group compared to the control group. Western blot analysis was performed to confirm whether these transcriptional alterations translated into changes at the protein level. Consistent with transcriptomic data, the relative protein expression of *CP, SLC7A11, GSTM1, and SLC40A1(FPN)* was markedly downregulated (P<0.05) in duodenal tissues in the treatment group (**Figures 4F-I**). This concordance between RNA-seq, mRNA, and protein expression strongly supports the involvement of ferroptosis in the observed pathological changes. Collectively, these findings indicate that OTA induces ferroptotic cell death in the duodenum primarily through the disruption of iron homeostasis, oxidative-antioxidant imbalance, and redox dysregulation. Quantification of the mean fluorescence intensity confirmed a significant decrease in *SLC7A11* signal in the treatment group compared to the controls, consistent with the mRNA and protein data (**Figures 5E-F**). Given that *SLC7A11* imports cystine for glutathione synthesis and thereby limits lipid peroxidation, its suppression is expected to compromise antioxidant capacity and sensitize cells to ferroptotic death. Together with RNA-seq, qRT-PCR, and western blot analyses showing the downregulation of *SLC7A11* and other ferroptosis-related genes, the IF results provide spatial evidence that OTA directly suppresses *SLC7A11* expression within the duodenal epithelium. This coordinated loss of *SLC7A11*-mediated cystine uptake and antioxidant defense, accompanied by iron and redox imbalance, strongly supports OTA-induced ferroptosis as the central mode of cell death in the duodenum.

### 3.5 OTA reshapes cecal microbiota homeostasis

**Figure 6:**
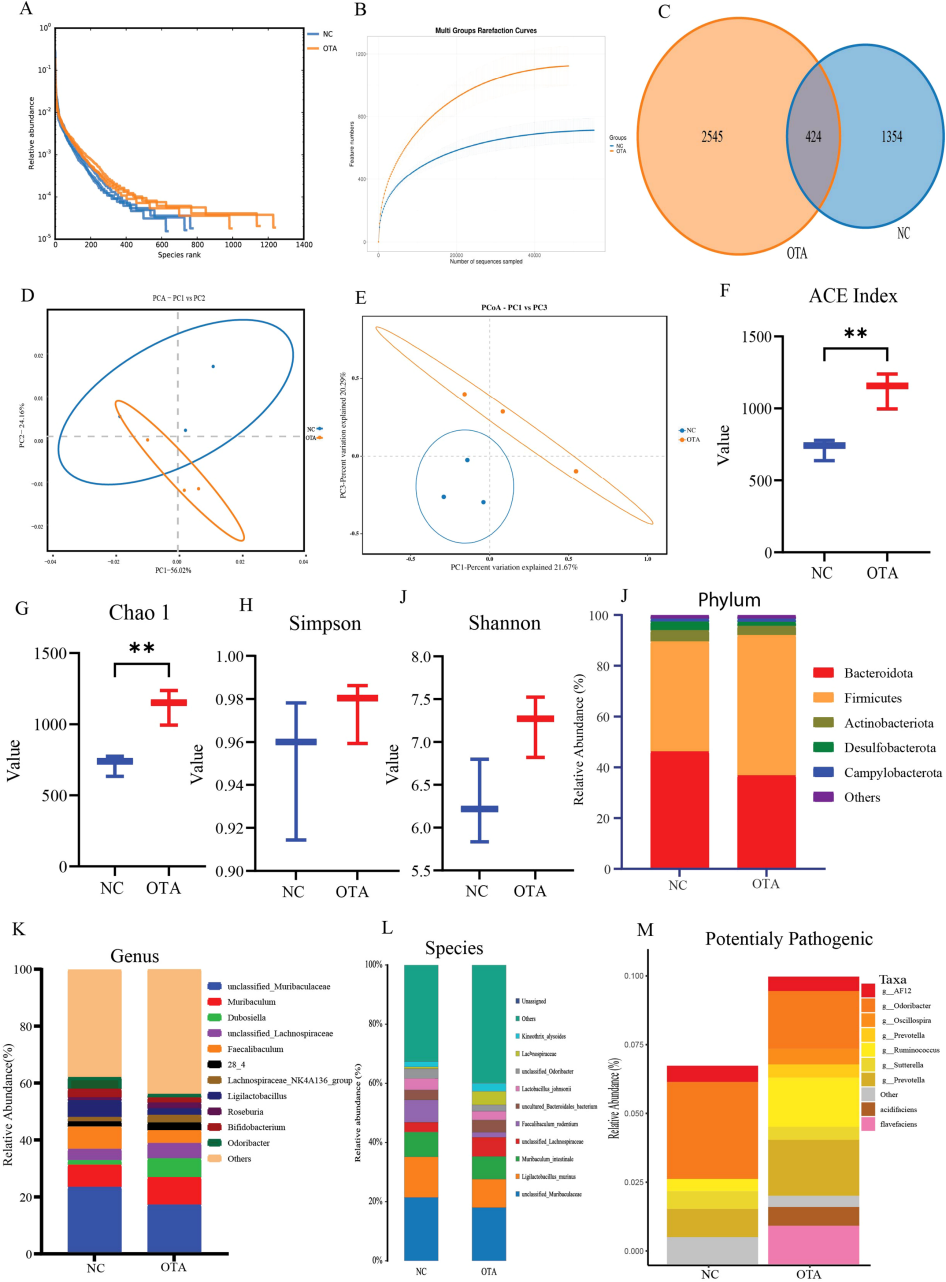
OTA induced gut microbiota dysbiosis and ferroptosis by elevating potentially pathogenic microbes in the treatment group **(A)** Relative abundance of different microbial communities in different samples **(B)** Rarefaction curve based on feature counts for microbes **(C)** Venn diagram for the number of OTUs in the control(NC) and treatment (OTA) groups **(D-E)** alpha and beta diversity of microbial community **(F-I)** Diversity indices for ACE,Chao1, Simpson, and Shannon **(J-L)** Changes in the gut microbiota subjected to OTA treatment at the phylum, genus, and species levels **(M)** Potentially pathogenic bacterial relative abundance changes in the control and treatment groups. All data are presented as mean ± SEM(n=3), with statistical significance indicated as follows: ^ns^P>0.05, **P<0.01.

16S rRNA sequencing of the cecal chyme microbiome showed that OTA exposure markedly altered the richness and structure of the intestinal microbiota. Overall, OTA increased alpha diversity and induced a clear separation of beta diversity between the control and treatment groups. The rank-abundance and rarefaction surveys for both groups tended toward saturation, indicating sufficient sequencing depth to capture the majority of microbial taxa present in the samples(**Figures 6A, B**) respectively. The Venn diagram revealed that, although the control and OTA groups shared a subset of operational taxonomic units(OTUs), OTA treatment led to the appearance of larger unique taxa, suggesting an OTA-driven reshaping of the microbial community(**Figure 6C**). Principal component analysis demonstrated distinct segregation between the control and OTA groups, reflecting marked differences in the overall microbial community composition(**Figure 6D, E**). In agreement with this, the ACE and Chao1 indices were significantly elevated in the OTA group, indicating increased species richness, whereas Simpson and Shannon showed a trend toward higher diversity after OTA exposure(**Figures 6F, I**). At the phylum level, OTA treatment altered the relative abundance of major bacterial groups, with noticeable changes in Bacteroidota, Firmicutes, and other major phyla compared to the controls(**Figure 6J**). At the genus level, OTA exposure led to a redistribution of dominant genera, including changes in unclassified Muribaculaceae, Lactobaccillus, and Bifidobacterium key taxa that are associated with intestinal homeostasis(**Figure 6K, L**). Importantly, OTA treatment was associated with an enrichment of bacteria classified as potentially pathogenic including *Oscillospira, Oscillibacter and Barnesiella* (**Figure 6M**), which promote inflammation and ferroptosis. This shift towards a more pathogenic microbial profile, together with increased richness and altered microbial community structure, suggested that OTA not only disrupts the microbiota composition but may also predispose the host intestine to inflammation, oxidative stress, redox imbalance, and barrier dysfunction. In parallel, 16S rRNA data showed increased richness, a clear shift in community structure, and enrichment of potentially pathogenic bacteria, which are known to promote ferroptosis by enhancing *ROS* generation, altering iron handling, and producing metabolites that suppress antioxidant systems, such as *SLC7A11-GSTM1* and *GCH1-BH4* mediated antioxidant. This pathogenic microbial shift, alongside increased diversity and community restructuring (**Figures 6A–M**), coincided with duodenal ferroptosis markers that suggested potential exacerbation of oxidative stress and barrier dysfunction through microbiota-duodenum axis.

### 3.6 Integrated microbiome and host duodenal transcriptome analysis links dysbiosis to ferroptosis injury mice intestine

Differential abundance analysis at the family level revealed that OTA exposure profoundly reshaped the intestinal microbial community (**Supplementary Table 4**). In particular, the relative abundance of Oscillospiraceae, Lachnospiraceae, Ruminococaceae, Peptosterptococcaceae, Bacteroidaceae, and several unclassified clostridial and Bacteroidales families was significantly increased in the OTA group compared to the controls, whereas members of Atopobiaceae and Eryipelatoclostridiaceae were markedly reduced (**Figure 5A**). Many of these enriched families include taxa associated with pro-inflammatory metabolism, bile acid transformation, and the production of metabolites that can aggravate oxidative stress and iron dysregulation in the intestine, providing a microbial context that favors ferroptotic injury of the duodenal epithelium. At the genus level, several taxa showed consistent and significant shifts further supporting OTA-induced dysbiosis. OTA treatment led to an overexpression of genera such as uncultured-Barnesiella, unclassified clostridia, Oscillibacter, Lachnospiraceae NK4A136 group, unclassified Lachnospiraceae, Romboutsia, Delftia, Colidextribacter, Flavonifractor, Family XIII UCG_001, and others, whereas Desulfovibrio, Eubacterium nodatum group, and some beneficial or commensal genera decreased (**Supplementary Table 5**) in abundance (**Figure 5B**). Many of these enriched genera, including Oscillibacter, Flavonifractor, Desulfovibrio, Barnesiella, and Colidextribacter, have been linked to impaired barrier function, heightened ROS production, or perturbed iron metabolism, thereby creating a luminal environment that can potentiate ferroptosis in host cells. The integration of host gene expression and redox homeostasis parameters, iron and *MDA* assessment, and microbiome data highlighted a tight coupling between ferroptosis-related pathways and specific microbial signatures. Ferroptosis is defined by the convergence of three processes: disruption of iron homeostasis with intracellular Fe accumulation, collapse of glutathione *(GSH)-GPX4* antioxidant capacity, and excessive lipid peroxidation, typically measured by malondialdehyde (*MDA*). The Pearson correlation heatmap (**Figure 5C**) showed that ferroptosis-associated markers *CP, SLC7A11, SLC40A1, GSTM1, HSPA5, FAR1, FLT3*, and *GCH1* and antioxidant indices *GSH, CAT, SOD*, and *T-AOC* was generally correlated with OTA-enriched genera, such as Desulfovibrio sp. ABHU1S, Oscillibacter sp.1_3, Ruminococcus lactaris, Colidextribacter, and Barnesiella sp. were significantly positively correlated with *HSPA5*, indicating a booster effect on antioxidant and ferroptotic resistance. Conversely, markers of oxidative damage and iron overload (*MDA* and *Fe*) exhibited a strong positive correlation with these OTA-associated genera, suggesting that the expansion of these bacteria is linked to reduced antioxidant defense, heightened lipid peroxidation, and iron accumulation in the duodenal mucosa, which are hallmarks of ferroptosis stress. Desulfovibrio and related anaerobes have been implicated in promoting mucosal oxidative stress and altering iron availability which may create pro-ferroptotic microenvironment that amplifies OTA-induced epithelial damage. This coordinated alteration of iron transport, glutathione metabolism, molecular chaperone activity, and gut microbial composition is highly consistent with the activation of ferroptosis in the duodenal mucosa under OTA challenge, providing mechanistic support for OTA-induced epithelial injury as ferroptosis-driven. Mental test-based network analysis further reinforced these relationships by demonstrating significant global correlations between microbiota structures and the ferroptosis-related gene set (**Figure 5D**). In this network, OTA-enriched families and genera were connected by strong edge arcs to downregulated genes such as *SLC7A11, CP, GSTM1*, and *SLC40A1*. These coordinated shifts indicate that OTA-induced dysbiosis is not merely a bystander phenomenon but is closely associated with the transcriptional reprogramming of iron handling, redox balance, and chaperone response in the host, jointly driving ferroptotic cell death in the duodenum. Collectively, these findings indicate that OTA exposure orchestrates a multi-layered ferroptosis response in duodenal mucosa. On the host side, toxins simultaneously augment iron and lipid peroxidation, deplete the core cystine-glutathione axis, and suppress iron export machinery while eliciting a compensatory HSPA5 mediated stress response that is insufficient to restore redox equilibrium. On the microbial side, OTA favors a consortium of taxa whose abundance is tightly associated with iron overload and oxidative damage and is inversely associated with ferroptosis-protective genes and antioxidant capacity. This convergence of biochemical markers, gene expression signatures, and microbiota-host correlation provides strong mechanistic support for the central role of ferroptosis in epithelial damage.

## 4 Discussion

OTA is a harmful mycotoxin, and its contamination during the transportation and storage of food and feed is challenging. It has been detected in 50% of livestock and poultry feed ingredients contaminated with OTA, posing a significant threat to human and animal health (61–63; R. 1). It has been observed that OTA is readily absorbed in most livestock from the duodenum and proximal jejunum (64). This study provides compelling evidence that OTA induces ferroptosis-like injury in the mouse duodenum through coordinated disruptions in redox balance, iron homeostasis, and gut microbiota composition. These findings extend previous observations of OTA toxicity beyond the kidney and liver to the proximal small intestine, where the epithelium serves as the primary exposure site (S. 1, 4). The duodenum, covered by epithelial cells, is considered the host’s largest defense barrier between the host and the external environment (65). The duodenal epithelium is the site of OTA exposure, and its impact on duodenal integrity and microbial dysbiosis regulating ferroptosis through duodenom transcriptome and cecal chyme microbiome is still unclear.

Furthermore, this study demonstrates that OTA exposure induces ferroptosis-like intestinal injury in mice, characterized by iron accumulation, lipid peroxidation, impaired antioxidant defenses, and disruption of the gut microbiota, thereby regulating ferroptosis. The integration of duodenal transcriptomics with cecal microbiome profiling highlights a bidirectional gut microbiota-duodenum axis in which dysbiosis amplifies redox imbalance and ferroptosis vulnerability (**Figures 5C, D**). This is consistent with earlier studies showing that mycotoxins, such as zearolenone, trigger intestinal ferroptosis via the system *GSH-GPX4* axis (66). OTA in current study significantly reduced *GSH, T-AOC, SOD*, and *CAT* (**Figures 1C-F**) as stated in previous studies (H. 67, 68). Increased *MDA* levels (69, 70) and tissue iron levels (**Figures 1G, H**) fulfilling the core biochemical criteria for ferroptotic cell death in the duodenum. Similar ferroptosis-related changes have been reported for OTA intestines with mitochondrial shrinkage, diminished cristae, rupture of cell membranes, cytoplasmic reticulum disruption (**Figure 3** A), and iron-dependent lipid peroxidation, supporting the hypothesis that OTA is a potent ferroptosis inducing toxin across organs (71). The pronounced reduction in villus height, increased crypt depth, and loss of goblet cells observed (**Figures 2B-E**) mirror the intestinal remodeling reported for other toxins as well as OTA toxin and stress models, where reduced villus height/crypt depth is closely linked to impaired digestion, absorption, and growth performance (19).

Goblet cells are central to mucus production and immune tolerance, and their depletion has been associated with mucosal inflammation and breakdown of epithelial defense, consistent with the *LPS* level detected after OTA exposure. The strong *4-HNE* immunopositivity in the OTA-treated duodenum (**Figure 2F**) further parallels reports that ferroptosis-related lipid peroxidation products accumulate in damaged intestinal tissues (72), reinforcing the interpretation that histological lesions reflect ferroptosis (73). By intersecting duodenal DEGs with a curated ferroptosis database, this study identified a focused panel of ferroptosis-associated genes, including *SLC7A11*,*GSTM1*,*CP*, and *SLC40A1*, that were consistently downregulated at both the mRNA and protein levels, in agreement with the central role of cystine import and iron export pathways in constraining ferroptosis (74). Similar bioinformatics strategies have linked ferroptosis gene sets with osteoarthritis, with enrichment of *ROS* metabolism and lipid handling pathways, indicating that OTA-induced transcriptomic patterns fit within a broader ferroptosis-related regulatory network. The enrichment of GO terms related to response to hydrogen peroxides, superoxides, *LPS*, and lipid metabolism, together with KEGG pathways such as fat digestion and absorption, *PPAR* signaling, and *NF-Kβ* signaling, echoes previous multi-omics studies in the intestine and joints that connect oxidative stress and ferroptosis at the system level (Z. 67, 75).

**Figure 7:**
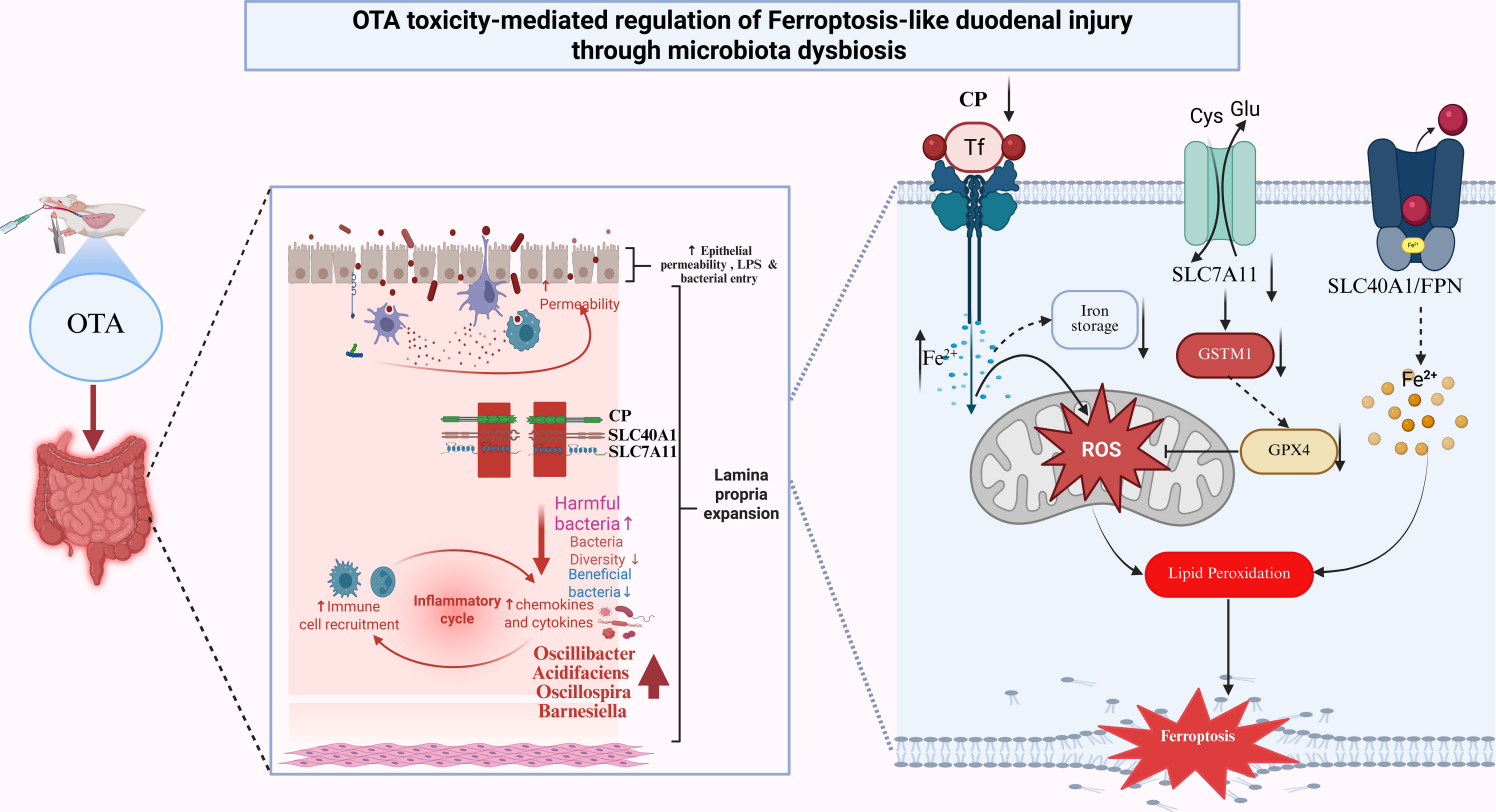
Schematic representation of OTA toxicity-mediated regulation of ferroptosis-like duodenal injury through microbiota dysbiosis and duodenal transcriptome.

While 16S rRNA sequencing revealed OTA-driven dysbiosis, with increased alpha diversity, Bacteroidota enrichment, and shifts in genera such as Oscillibacter and Barnesiella, alongside pathogenic taxa proliferation. Correlation networks linked these microbes to ferroptosis markers, suggesting that dysbiosis amplifies epithelial vulnerability through ROS-promoting genera and impaired barrier function, consistent with OTA altering Firmicutes/Bacteroidetes ratios in previous rodent models (76). OTA-treated mice in this study exhibited increased alpha diversity, clear beta-diversity separation, and enrichment of potentially pathogenic taxa in the treated group, resembling the dysbiosis pattern described for ZEA-exposed rats. *Phocaeicola acidifaciens* and *Ruminococcus flavefaciens* (**Figure 6M** pink bars) showed OTA-specific increases while *P. acidifaciens* protects gut homeostasis, dysbiotic expansion promotes mucus degradation and colitis. While *R. flavefaciens* alterations link to ROS/barrier dysfunction, plausibly enhancing ferroptosis via oxidative stress (77–79). In inflammatory and degenerative models, the Firmicutes/Bacteroidetes ratio and key genera, such as Lachnospiraceae, Oscillospiraceae, Ruminococcaceae, and Bifidobacterium, were significantly altered (80). The observed correlation in this study between differential bacterial genera and ferroptosis-related parameters (iron,*MDA*, and redox indices) and expression of *SLC7A11,GSTM1,CP*, and *SLC40A1* supports a model in which OTA-driven dysbiosis forms a vicious cycle with epithelial ferroptosis, collectively destabilizing barrier integrity and systemic homeostasis (81). This microbiota-duodenum axis forms a feedback loop in which ferroptotic damage further destabilizes the microbial ecology. These findings are consistent with interventional studies in which microbiota strategies attenuate ferroptosis, suggesting possible translational avenues (82). Taken together, these findings position OTA as a prototypical dietary toxin that couples disruption of iron and redox homeostasis with microbiota remodeling to drive ferroptosis-mediated intestinal (**Figure 7**) injury, extending previous reports of OTA-induced ferroptosis in the renal and hepatic systems to the duodenum. By integrating histology, ultrastructure, redox biochemistry, ferroptosis-focused transcriptome, and microbiome ecology, this study provides mechanistic evidence that targeting both epithelial ferroptosis pathways and microbiota-driven signals may be necessary for effective prevention of OTA-associated enteropathy in humans and livestock.

## 5 Conclusions

This study elucidates that OTA induces ferroptosis-like damage in the mouse duodenum by disrupting iron homeostasis, redox balance, and gut microbiota composition, as demonstrated through integrated transcriptomic and microbiome analyses. OTA administration resulted in decreased body weight, elevated serum *LPS*, increased duodenal *MDA* and iron levels, and depletion of essential antioxidants, including *GSH, T-AOC, CAT*, and *T-SOD*, thereby fulfilling the established biochemical criteria for ferroptosis. Histological alterations included villus atrophy, crypt hyperplasia, goblet cell depletion, and pronounced *4-HNE* immunoreactivity. Transcriptomic profiling identified 769 DEGs, with 26 overlapping ferroptosis genes (FerroDb) enriched in *ROS* response and lipid metabolism. Moreover, 16S RNA sequencing indicated an OTA-induced increase in alpha diversity, shifts in beta diversity, enrichment of Bacteroidetes, and proliferation of pathogenic taxa (Oscillibacter and Barnesiella), with correlation networks linking dysbiotic genera to ferroptosis markers, iron overload, and antioxidant collapse. This reveals a microbiota-duodenum axis in which dysbiosis exacerbates epithelial ferroptosis in a bidirectional feedback loop. Collectively, OTA acts as a potent inducer of duodenal ferroptosis via cystine-glutathione axis failure and pro-oxidant microbial modeling, advancing the understanding of mycotoxin enteropathy and highlighting microbiota-targeted strategies for mitigating toxicity in food safety and human and animal health.

## Data Availability

All raw sequencing data generated in this study were deposited in the NCBI Sequence Read Archive (SRA) under the BioProject accession number (PRJNA1397339). The dataset included RNA-seq and 16S rRNA microbiome sequencing, with each biological replicate submitted as a distinct BioSample.
