# Supplementary material for: Integrative transcriptome and microbiome analysis reveals ferroptosis-driven duodenal damage caused by Ochratoxin A in mice
Source: Front Immunol. 2026 Apr 13;17:1804647. doi: 10.3389/fimmu.2026.1804647 (PMC13110954; doi:10.3389/fimmu.2026.1804647)
Supplement: Supplementary file 1 [file Table1.docx]

Supplementary Table 1. Summary of data from RNA-seq

| Sample | Obtained Reads | Q 20 | Q 30 | Mapped reads | Total  mapped rate1 | Uniquely mapped rate2 |
| --- | --- | --- | --- | --- | --- | --- |
| RNA-Control-Rep1-Duodenum | 21934253 | 99.19 | 97.26 | 21509752 | 98.06% | 81.14% |
| RNA-Control-Rep2-Duodenum | 22127445 | 99.21 | 97.32 | 21741500 | 98.26% | 81.43% |
| RNA-Control-Rep3-Duodenum | 23307148 | 99.00 | 96.65 | 22930577 | 98.38% | 81.60% |
| RNA-Ochratoxin A-Rep1-Duodenum | 22452686 | 99.27 | 97.57 | 22140857 | 98.61% | 79.35% |
| RNA-Ochratoxin A-Rep2-Duodenum | 22094971 | 99.07 | 96.84 | 21723730 | 98.32% | 73.19% |
| RNA-Ochratoxin A-Rep3-Duodenum | 24167358 | 99.10 | 96.90 | 23747218 | 98.26% | 77.74% |

^1^Total mapped rate = number of clean reads and the ratio that matched multiple position in the genome.

^2^Uniquely mapped rate = number of clean reads and the ratio that matched only one position in the genome.
