# Supplementary material for: Integrative transcriptome and microbiome analysis reveals ferroptosis-driven duodenal damage caused by Ochratoxin A in mice
Source: Front Immunol. 2026 Apr 13;17:1804647. doi: 10.3389/fimmu.2026.1804647 (PMC13110954; doi:10.3389/fimmu.2026.1804647)
Supplement: Supplementary file 2 [file Table2.docx]

Supplementary Table 4. List of the primer sequences

| **Name** | **Sequences** |
| --- | --- |
| Mouse-SLC7A11-1-F | CAGGCATCTTCATCTCCCCC |
| Mouse-SLC7A11-1-R | AGCCAGCAAAGGACCAAAGA |
| Mouse-CP-F | CGTCTACCCTGACAACACCA |
| Mouse-CP-R | GAGGTCCTATGAGTCCTGATGC |
| Mouse-GSTM1-F | CACCTGGATGGAGAGACAGAG |
| Mouse-GSTM1-R | TCACCACCTTTAGACTCAGGC |
| Mouse-SLC40A1-1-F | AATGTGGCCTTGTTCGGACT |
| Mouse-SLC40A1-1-R | GGACACTGGCTCCACATTCA |
| Mouse-GAPDH-1-F | GGAGAGTGTTTCCTCGTCCC |
| Mouse-GAPDH-1-R | TTTGCCGTGAGTGGAGTCAT |
